# Supplementary material for: Recurrent WNT pathway alterations are frequent in relapsed small cell lung cancer
Source: Nat Commun. 2018 Sep 17;9:3787. doi: 10.1038/s41467-018-06162-9 (PMC6141466; doi:10.1038/s41467-018-06162-9)
Supplement: Supplementary file 1 — Supplementary Information [file 41467_2018_6162_MOESM1_ESM.pdf]

Recurrent WNT Pathway Alterations are Frequent in Relapsed Small Cell Lung Cancer

Wagner, et al.

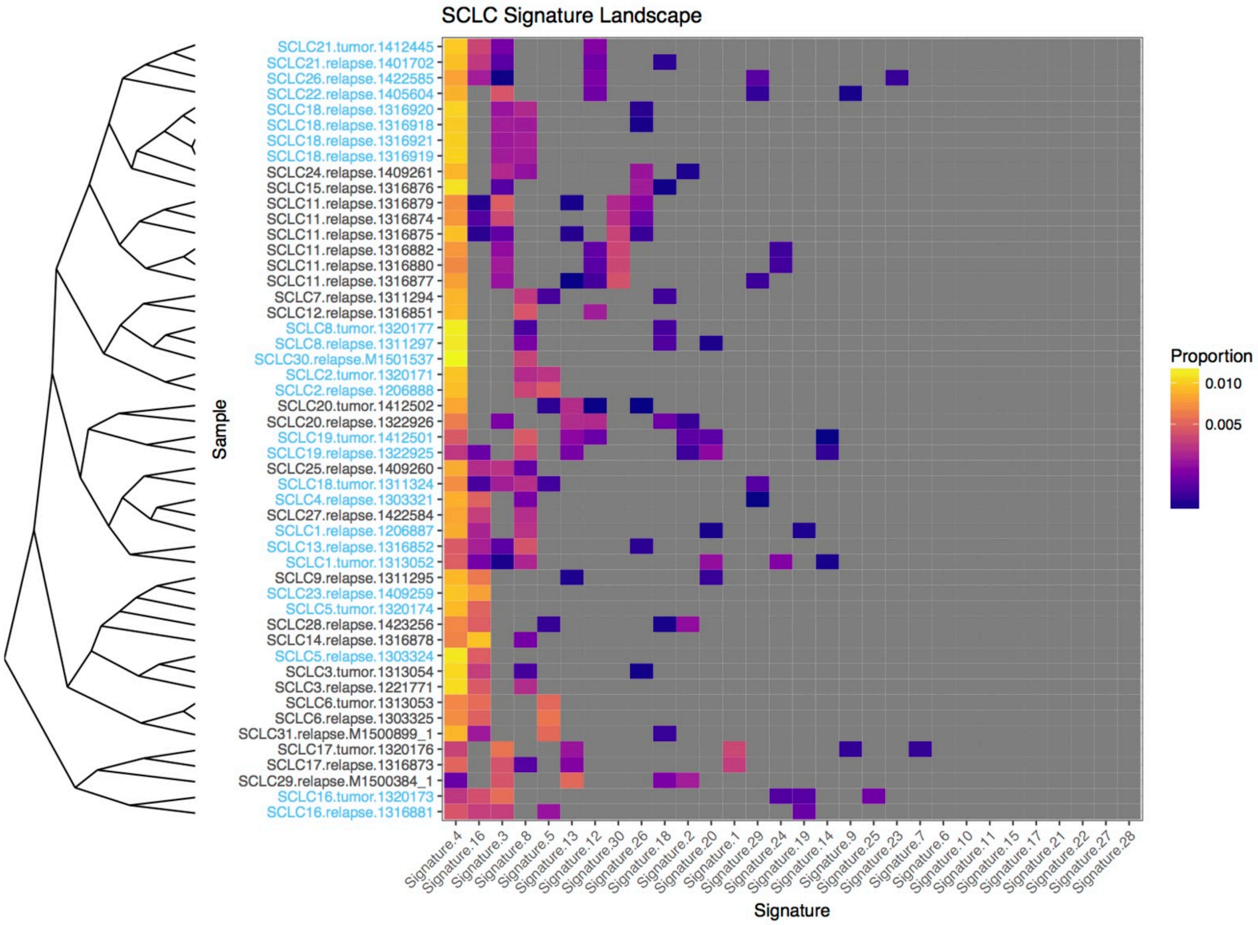

Supplementary Figure 1 COSMIC Mutation Signatures of SCLCs.

Proportion of mutations across the exome resembling 30 known COSMIC mutation signatures (https://cancer.sanger.ac.uk/cosmic/signatures) in relapsed and paired treatment-naive SCLC samples are represented as a heatmap. Limited stage SCLC samples exposed to irradiation are denoted with blue text. As expected, tobacco smoking associated mutation signature (signature 4) was the predominant mutation signature across all samples. The pancreatic tumor platinum response associated mutation signature (signature 3) accounted for a small, non-zero proportion of mutations in a subset of samples. Cohort wide, all signatures except signature 4 were less prevalent than the residual "unknown" signature (not shown). Color scale increases from low proportion of signature (blue) in sample to high (yellow).

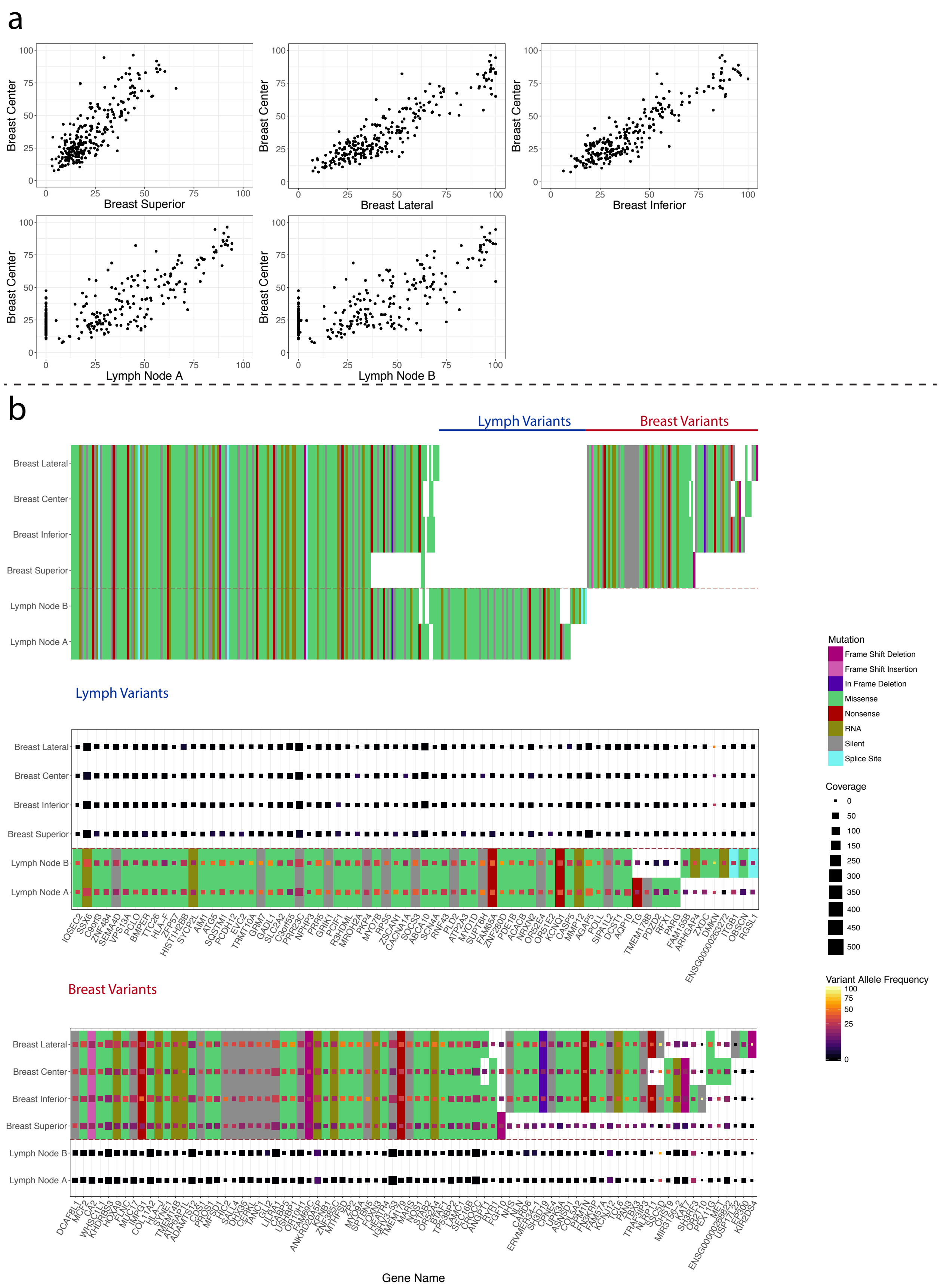
a
Breast Center
Breast Superior
Breast Lateral
Breast Inferior
Lymph Node A
Lymph Node B
0
25
50
75
100
b
Lymph Variants
Breast Variants
Breast Lateral
Breast Center
Breast Inferior
Breast Superior
Lymph Node B
Lymph Node A
Mutation
Frame Shift Deletion
Frame Shift Insertion
In Frame Deletion
Missense
Nonsense
RNA
Silent
Splice Site
Coverage
0
50
100
150
250
300
350
400
450
500
Variant Allele Frequency
100
75
50
25
0
Gene Name

Supplementary Figure 2. Spatial and inter-tumoral heterogeneity in relapsed SCLC.

(A) Breast metastasis for SCLC11 was quartered (superior, inferior and lateral) and sequenced as separate samples along with one lymph node metastasis that was halved (halves A and B). Variant allele fractions (VAF) of manually reviewed variants for each tissue are shown in a pairwise fashion. (B) Mutation landscape is shown for SCLC11 samples. VAFs and coverage amounts (COV) are shown for mutations specific to individual groups of sequenced tissue. Variants were identified as tissue specific by observation of the variant in ≥50% of samples of only one tissue. Variant allele frequency of coverage boxes increases from blue (0% of reads describe variant) to yellow (100% of reads describe variant).

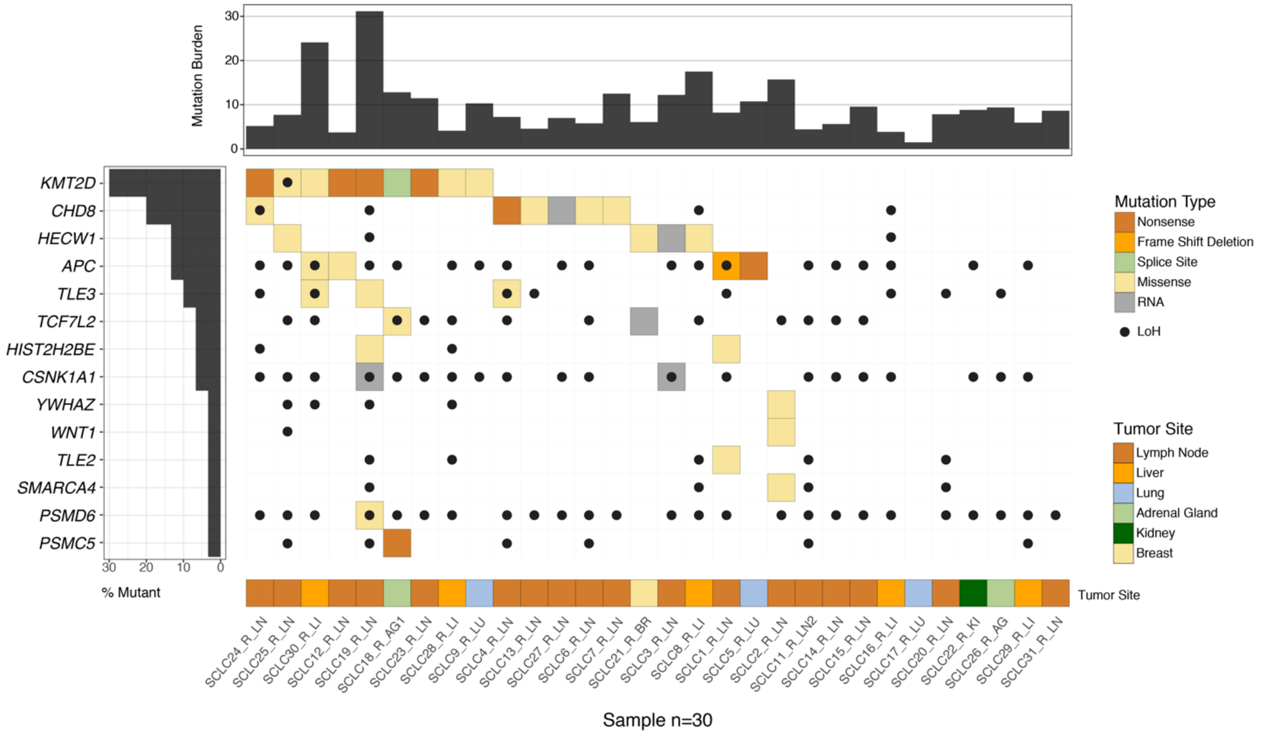

Supplementary Figure 3. Relapse-acquired WNT signaling genes.
Mutations in canonical WNT signaling pathway genes with either relapse-acquired mutations or genes that were unaltered in treatment-naive samples, across all 30 relapse SCLC samples. Mutation burden (mutations/mega-base pair) is displayed on the x-axis panel (top), and cohort mutation percentage by gene is displayed on the y-axis panel (left). Coloring denotes mutation type (top legend, center panel) and tumor site (bottom legend, bottom panel). Black dots on sample-gene squares indicate loss of heterozygosity (LoH) of that gene in that sample. Each column represents an individual sample (listed at bottom). TN=treatment-naive, R=relapsed, LN=lymph node, AG=adrenal gland, LU=lung, BR=breast, KI=kidney, LI=liver.

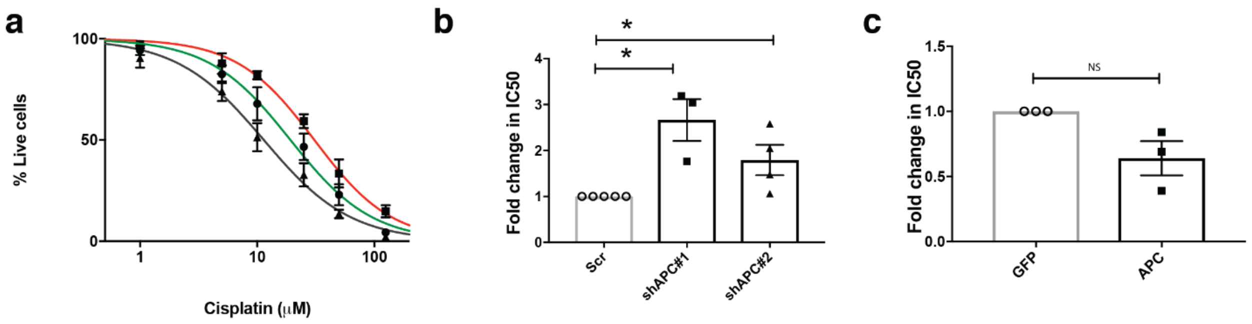

Supplementary Figure 4. Loss of APC is associated with cisplatin resistance
(a) Cell survival curves representing resistance to cisplatin in H1694 cells following stable APC knockdown with constructs shAPC#1 (red) and shAPC#2 (green). Control cells (black) express shRNA targeting a scramble (Scr) sequence. **(b)** Fold changes in cisplatin IC50 between H1694 cells expressing shAPC#1, shAPC#2 and shScr (control). **(c)** Fold changes in cisplatin IC50 following overexpression of wildtype APC in shAPC#2 expressing H1694 cells, compared to cells overexpressing green fluorescent protein (GFP). All experiments were performed in biological triplicate, while cisplatin IC50 for shAPC#2 cells was determined from n=4 experiments. IC50 values were compared using ratio-paired t-tests. (*)=$p<0.05$, ns=non-significant. Error bars on box plots and dose-response curves are mean +/- standard error.

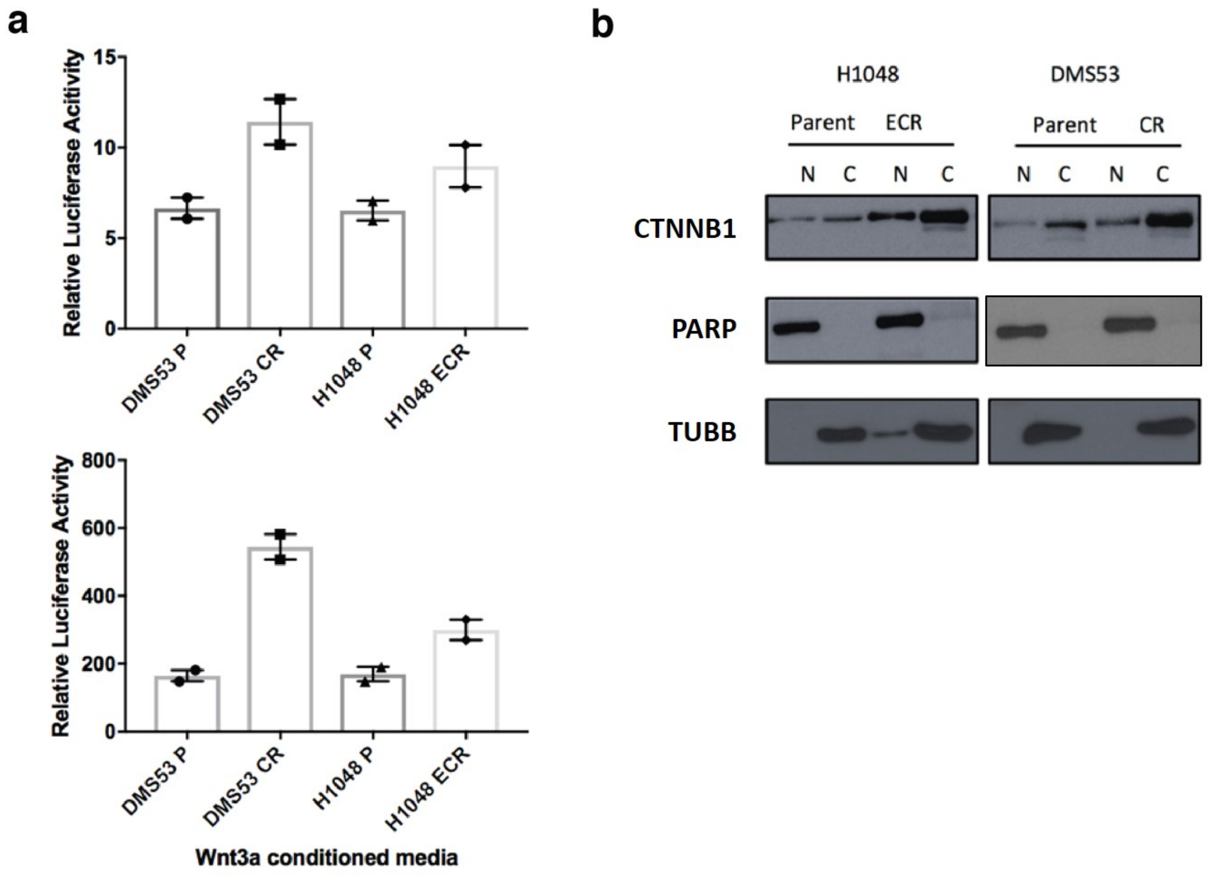

Supplementary Figure 5. Chemotherapy resistant human SCLC cell lines show increased WNT activity. **(a)** Increased level of TCF/LEF (TOPFlash) reporter activity in chemoresistant cells, when compared to their parental chemo-naive counterparts cultured either in normal media or Wnt3a-conditioned media. Box plots show mean +/- standard error. Experiments were performed in duplicate.**(b)** Representative western blot for indicated cell lines with increased nuclear (N) and cytoplasmic (C) expression of β-catenin (CTNNB1) in resistant cell lines. PARP and β-tubulin (TUBB) serve as nuclear and cytoplasmic loading controls, respectively. CR = cisplatin resistant, P = parental, ECR = etoposide/cisplatin resistant. Error bars on box plots are mean +/- standard error.

Uncropped Western Blot
(Figure 4e)

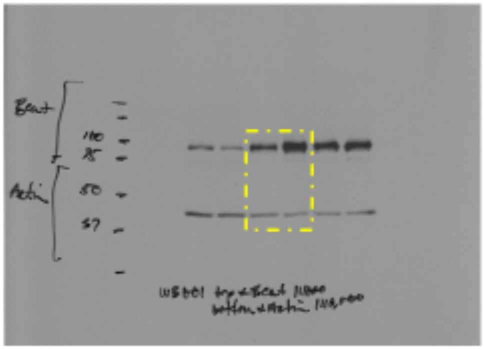

Band sizes for Molecular weight marker are 250, 150, 100, 75, 50, 37, and 25kD
(Precision Plus Protein Dual Color Standards, BioRad, #1610394)

Uncropped DNA Gel for Surveyor Assay
(Figure 4d)

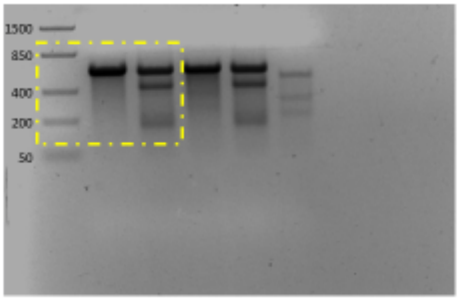

Band sizes for DNA ladder are shown in base pairs
(FastRuler Low Range DNA Ladder)

Uncropped Western Blot
(Supplementary Figure 5b)

CTNNB1

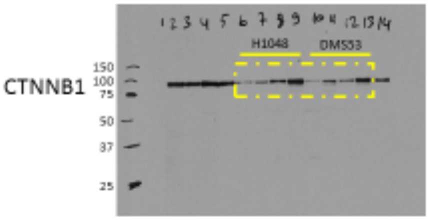

PARP

NEMO

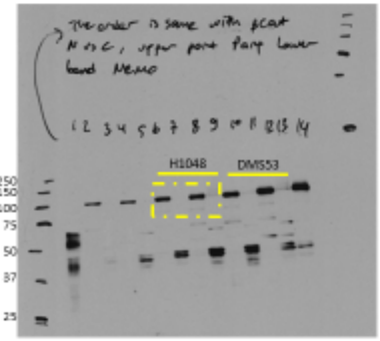

PARP, long exposure

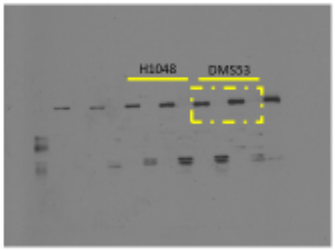

PARP, short exposure

Uncropped Western Blot
(Supplementary Figure 5b)

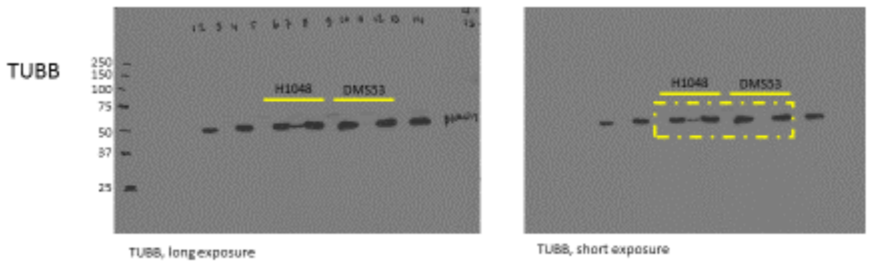

Supplementary Figure 6. Uncropped western blot images for for experiments with NCI-H82, NCI-H1048 and DMS53 cells.
