# Supplementary material for: Recurrent WNT pathway alterations are frequent in relapsed small cell lung cancer
Source: Nat Commun. 2018 Sep 17;9:3787. doi: 10.1038/s41467-018-06162-9 (PMC6141466; doi:10.1038/s41467-018-06162-9)
Supplement: Supplementary file 3 — Description of Additional Supplementary Files [file 41467_2018_6162_MOESM3_ESM.pdf]

Description of Additional Supplementary Files

File Name: Supplementary Data 1
Description: *Sample Details:* Clinical characteristics, treatment history, and sample information for the relapsed SCLC cohort.

File Name: Supplementary Data 2
Description: *Immunohistochemical Features of SCLC Samples*: pathologist scoring of histopathological stains for various disease-relevant markers in cohort samples.

File Name: Supplementary Data 3
Description: *Significantly Mutated Genes*: list of genes identified as significantly recurrently mutated in relapsed SCLC cohort by MuSiC analysis.

File Name: Supplementary Data 4
Description: – *Significant Copy Number Alterations*: list of loci and constituent genes with significant copy number alterations in relapsed SCLC cohort by GISTIC analysis.

File Name: Supplementary Data 5
Description: *Relapse Genes*: list of genes identified as relapse-acquired (present only in relapse for primary-relapse pairs) or relapse-only (present only in relapsed samples, not primaries).

File Name: Supplementary Data 6
Description: *Pan-cancer Gene List*: list of previously identified significant pan-cancer genes, and the number of mutations observed across primary, paired relapse, and all relapse tumors.

File Name: Supplementary Data 7
Description: – *Canonical WNT Pathway Gene List*: list of previously identified WNTsignaling genes, and the number of mutations observed across primary, paired relapse, and all relapse tumors.

File Name: Supplementary Data 8
Description: *Sample Work Orders*: a brief description of sample type and mapping of samples to work orders (Supplementary Data 9).

File Name: Supplementary Data 9
Description: *Work Order Details*: a description of sequencing protocols and characteristics related to each work order.

File Name: Supplementary Data 10
Description: *All SNVs and Indels*: somatic single nucleotide variants and small insertions and deletions observed across all tumor samples.

File Name: Supplementary Data 11
Description: – *Significantly Mutated Gene Loss of Heterozygosity Calls*: loss of heterozygosity calls as determined by zygosity score thresholding across all significantly mutated genes (Supplementary Data 3).

File Name: Supplementary Data 12
Description: *sGSEA Enrichment Scores*: enrichment scores across 4 distinct gene expression signatures for RNA samples from this and previous studies.
